# The Effects of Resistance Training on Physical Fitness and Neuromotor-Cognitive Functions in Adults With Down Syndrome

**DOI:** 10.3389/fresc.2022.927629

**Published:** 2022-06-21

**Authors:** Emily M. Post, William J. Kraemer, Madison L. Kackley, Lydia K. Caldwell, Jeff S. Volek, Barbara N. Sanchez, Brian C. Focht, Robert U. Newton, Keijo Häkkinen, Carl M. Maresh

**Affiliations:** ^1^Department of Exercise Science, Ohio Dominican University, Columbus, OH, United States; ^2^Department of Human Sciences, The Ohio State University, Columbus, OH, United States; ^3^Exercise Medicine Research Institute, and School of Medical and Health Sciences, Edith Cowan University, Joondalup, WA, Australia; ^4^Kinesiology, Health Promotion and Recreation, University of North Texas, Denton, TX, United States; ^5^Neuromuscular Research Center, Biology of Physical Activity, Faculty of Sport and Health Sciences, University of Jyväskylä, Jyväskylä, Finland

**Keywords:** exercise, cognition, motor skill, Trisomy 21, strength, special populations

## Abstract

Adults with Down syndrome are an underserved population at high risk for a host of different pathologies from aging and lack of activity.

**Purpose:**

To examine the effects of a 10-week resistance training program on measures of motor behavior, cognitive function, mood, and physical fitness.

**Methods:**

Participants (*n* = 11) were men and women clinically diagnosed with Down syndrome (age: 25.8 ± 6.4 years; height: 151.5 ± 8.3 cm; weight: 67.5 ± 13.0 kg; IQ: 58.3 ± 19.7 units). After familiarization of testing procedures, subjects performed The Arizona Cognitive Test Battery for Down Syndrome, TGMD-2, lower and upper body strength assessments, and body composition via DXA testing, while parental guardians completed cognitive and mood survey assessments (Cognitive Scale for Down Syndrome, Behavioral Rating Inventory of Executive Function, NiSonger Child Behavior Rating Form, Scales of Independent Behavior-Revised, Child Eating Behavior Questionnaire, Social Communication Questionnaire, and Mood and Feelings Questionnaire) at pre and post 10 weeks of periodized resistance training.

**Results:**

Significant (*P* ≤ 0.05) improvements in locomotor skills and object control skills were observed post-training. Both locomotor skills (e.g., sprint, gallop, leaping, broad jump) and object control skills (e.g., baseball catch, underhand roll, basketball dribble) were all significantly improved. Facets of cognitive performance significantly improved, specifically executive function and visuospatial working memory capacity, and frontal lobe activity. Mood disturbances significantly decrease. All aspects of physical strength and endurance were improved, i.e., leg press, bench press, sit-ups, push-ups, and chair sit-to-stand post-training. Lean tissue mass was significantly increased post-training.

**Conclusion:**

This study dramatically demonstrates that life enhancements for individuals with Down syndrome are achievable with a properly designed resistance training program.

## Introduction

The incidence of Down syndrome is estimated to be between 1 in 1,000 to 1 in 1,100 live births worldwide. Approximately 1 out of every 700 children in the United States is born with Down syndrome (i.e., Trisomy 21), making Down syndrome the most common chromosomal disorder (1). Even so, adult individuals with Down syndrome are a dramatically underserved population at very high risk for a host of different pathologies from more rapid aging effects, cognitive deficits, and accidental injuries. Physical activity has been shown to be an important intervention for the aforementioned effects in typically developed adults without Down syndrome. Still, little research has been done to examine the impact for people with Down syndrome (2). Initial studies in resistance training for individuals with Down syndrome showed that this modality holds great promise for positive effects in both fitness and performance (2). Yet this area of research in resistance training has been highly understudied, if not abandoned, for almost 20 years or more despite the amazing potential for discoveries that could dramatically impact the quality of life for adults with Down syndrome. Thus, our research groups endeavored to bring this line of research back into the forefront of exercise science by undertaking a resistance exercise training study in young adults with Down syndrome.

Numerous studies have explored aspects of brain function and genetics with Down syndrome in relationship to Alzheimer's disease (3–5). Still, there currently is no literature examining resistance training's effects on cognitive performance specifically and very little regarding exercise in general. However, there is limited evidence in other populations of individuals with intellectual handicaps showing improvements in cognition following resistance exercise, which may promote a better anti-inflammatory balance helping to reduce amyloid plaques (6, 7). It was evident that the range of benefits of resistance training for individuals with Down syndrome has been relatively unexplored. Yet, its proven efficacy in other populations who are intellectually compromised supports the critical need for this investigation.

Feelings and mood states are also vital for a positive outlook and interactions with other people. Resistance training has been shown to create a positive outlook on exercise and decrease incidences of depression for people with Down syndrome (8). Furthermore, overall cognitive function and emotional and mental state are mediated by positive mood states after exercise in healthy adults, allowing mechanistic possibilities for this same improvement for adults with Down syndrome (6, 9). Our group's prior anecdotal experiences with family members and colleagues who have relatives with Down syndrome have supported the concept that resistance exercise is a modality that is well-received and enjoyed. Thus, it is imperative and long overdue that further scientific support is provided to make more evidence-based data for the use of the modality.

Additionally, physical activity levels in individuals with Down syndrome are lower and typically decrease over time at a greater rate than individuals without Down syndrome, leading to higher rates of metabolic disease and obesity (10). Increased exercise capacities can have immense impacts on health and work productivity. People with Down syndrome also typically have lower strength and less balance, which can cause more accidents and a decline in health overall (11). The higher prevalence of accidents and falls in individuals with Down syndrome decreases the quality of life and is problematic for parents/guardians. Resistance training has been shown to ameliorate these difficulties, showing an overall benefit in strength gains, better balance, and motor function post resistance training in the population of people with Down syndrome (2, 12–15). While the body of literature for this population is small, the benefits are very provocative and again support the need for more study of this modality as a critical intervention.

We hypothesized that resistance training would improve cognition, motor function, and mood in young adults with Down syndrome. We hypothesized that a properly designed periodized resistance training program would improve physical strength and lean body mass in young adults with Down syndrome. If resistance training can significantly improve physical strength, body composition, bone density, mood, and cognitive performance, it would be a viable intervention to help improve the health status and quality of life for people with Down syndrome.

## Methods and Materials

This study was approved by The Ohio State University's Institutional Review Board for the use of human subjects in research. After all of the risks and benefits of the study were carefully explained to the participants and their parent/guardian, written informed consent was obtained from the parent/parental guardian and assent from the participant (if applicable) using an institutionally approved informed consent document following the UC Davis' policy and procedures for assessing capacity to consent for research used in special populations (16). Each participant understood that s/he had the freedom to drop out from the study or not perform any test if s/he did not feel they wanted to participate. The participant must have been able to understand how to perform the cognitive tests that have been specifically designed for this special population.

A single group model was utilized due to the fact that matching for various aspects of the study's dependent variables (i.e., cognitive impairment/function) is not feasible. This is due to the high variability of genotype penetration to a particular phenotype variable being studied. Thus, individuals with Down syndrome can demonstrate different inherent capabilities due to this phenomenon of genotype penetration of the extra 3rd gene on the 21st chromosome to a phenotypic characteristic or function. Thus, to determine the effects of the intervention we used familiarizations and a solid baseline stability showed that all measures used in this study had ICCRs of *P* ≥ 0.75.

### Study Participants

**Figure 1:**
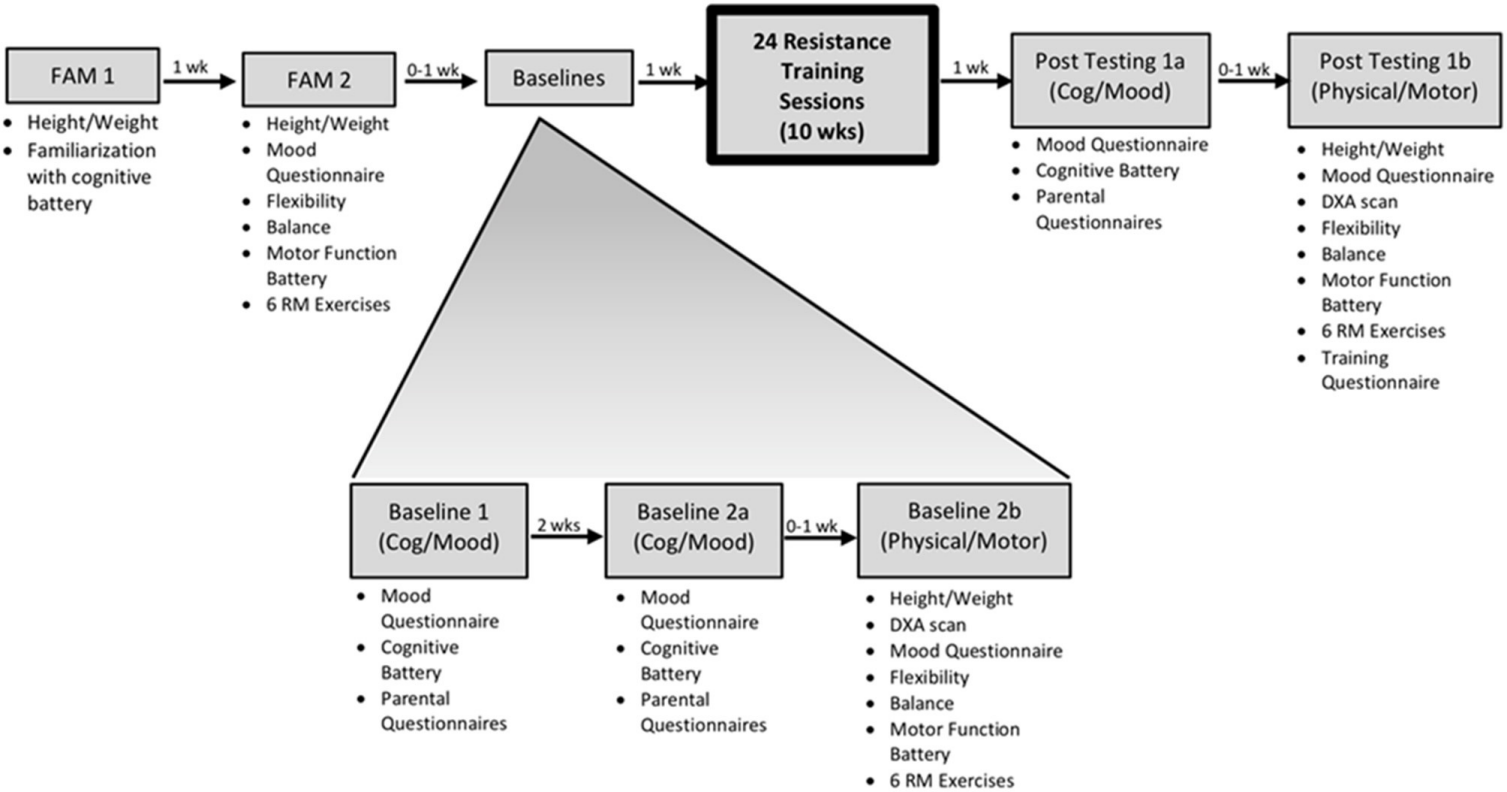
Overview of the experimental design. Individual familiarization visits were performed for both the cognitive and physical batteries to minimize any learning effects. The concept of a lack of control group was addressed by having reliability from multiple baseline visits and due to the fact that matching for IQ and functionality is not a valid design and is rarely employed with this population.

Participants were recruited through the Down Syndrome Association of Central Ohio. The participants (*n* = 11) were at least minimally active men (*n* = 6) and women (*n* = 5) and were between the ages of 18–37 years old, clinically diagnosed with Down syndrome (age 25.8 ± 6.4 years; height 151.5 ± 8.3 cm; weight 74.9 ± 23.4 kg; IQ: 58.9 ± 18.5 units). Down syndrome diagnosis and physician clearance for exercise were confirmed through the participant's primary physician. Participant characteristics at pre-testing are shown in Table 1. The research design is shown in Figure 1.

**Table 1:** Participants demographics at pre-testing (*n* = 11; *n* = 6 men and *n* = 5 women).

	**Mean ±SD**	**Minimum**	**Maximum**
Age (years old)	25.8 ± 6.4	18.2	36.1
Men	25.1 ± 7.4	18.2	36.1
Women	26.7 ± 5.7	19.7	32.9
Height (cm)	151.5 ± 8.3	141.8	163.7
Men	157.0 ± 6.7	148.2	163.7
Women	144.9 ± 4.1^*^	141.8	151.8
Weight (kg)	74.9 ± 23.4	51.8	134.8
Men	79.8 ± 28.9	55.4	134.8
Women	69.0 ± 15.9	51.8	85.7
IQ (units)	58.9 ± 18.5	10	79.0
Men	54.8 ± 24.3	10.0	79.0
Women	63.8 ± 7.9	53.0	74.0

The overall IQ range for this cognitive test for all individuals, as assessed by the Kaufman Brief Intelligence Test – 2nd edition (general population, intellectually impaired, etc.) typically ranges from 40 to 160 units (17). “*”, significantly different than the men of that same variable.

### Familiarization Visits

The familiarization period ensured that the participants could perform each test with full comfortability. Each participant completed two familiarization visits. The first familiarization visit (FAM 1) was to familiarize the participant with the full cognitive battery via Arizona Cognitive Test Battery for Down Syndrome (ACTB-DS), along with addressing IQ via the Kaufman Brief Intelligence Test – 2nd edition (KBITT-2). The second familiarization visit (FAM 2) involved the practice of flexibility (sit-and-reach test), physical strength (bench press and leg press 6-RM testing, 30-s push-ups, 30-s sit-ups, chair sit-to-stand), and motor function testing (TGMD-2) (18).

### Baseline Visits

Participants completed a total of 3 baseline visits (1, 2a, and 2b). Baseline visit 1 was approximately 1 week after FAM 1. Baseline 1 and 2a (2 weeks apart) were identical visits that consisted of cognitive battery assessments, mood questionnaires, and parental questionnaires. Baseline visit 2b consisted of physical measures (i.e., height/weight, DXA scan, sit-and-reach flexibility test, physical strength tests, and motor tests).

### Resistance Training Intervention

Working with this population of individuals does have its context for instruction and performance as the cognitive understanding of health performance benefits and other aspects we take for granted in the typical fitness populations are not present. Thus, it is vital to have a fun experience and individualized understanding of teaching exercise movements and promoting resistance training related to their enjoyment. Rewards are critical to the effectiveness of programming and individual relationships with each participant and their parents/guardians in this process. The resistance training program was also implemented with an intrinsic reward system shown to be necessary to motivate individuals to do their best, as this has typically been an effective teaching approach to task orient individuals with Down syndrome. Participants chose what music they wanted to listen to throughout the exercise session. They also received two movie tickets when they completed half of the resistance training visits and another two movie tickets when they fully completed the study.

A 10-week resistance training (RT) exercise protocol (24 supervised sessions) was implemented after the initial 2-week introductory phase (2 familiarization and 3 baseline sessions). The 10-week resistance training protocol consisted of light Repetition Maximum (RM) (12–15 RM), moderate (8–10 RM), and heavy (4–6 RM) for three sets. The introductory phase consisted of 12–15 RM at light and 8-10 RM at a moderate weight. Set number progressed from 1–3 sets by the end of the 2-week introductory phase. Teaching and demonstrating exercises took much attention and greater care using manual cuing and movement tracking as teaching exercises to many with Down syndrome is different due to mental imagery differences in motor translation from showing exercise movement to having the participant produce the movement. Participant toleration for workouts was noted during each session. Heavy sets were worked into the exercise programming after the initial 2-week period based on exercise tolerance. Changes in exercise volume depended on the individual's capabilities and assessments by the certified (CSCS) research team trainer. The resistance training exercises progressed after warm-ups from large to smaller muscle groups (e.g., leg press, bench press, leg curl, shoulder press, bicep curls, and variations to make it enjoyable each day). Exercises were performed with machines, free weights, resistance bands, and body weight alone to meet the individual loading needs in the different exercises (2). Although individualized on motor skill level and strength, all participants exercised the same groups of muscles for each of their training visits.

Participants were given longer rest periods between each set than the average adult and appropriate hydration breaks due to metabolic issues with individuals with Down syndrome and to reduce any symptoms of metabolic stress and common problems with thermoregulation and sweat gland dysfunction (19). Each resistance exercise training visit was between 45 and 60 min in duration. Heart rate monitors were worn during the exercise session and into recovery to determine stress levels, help the trainer maintain safety, and monitor exercise intensity (heart rate per session - mean ± SD; 104.0 ± 8.0 bpm; [Polar Heart Rate Monitor Kempele, Finland]). This average heart rate range would qualify individuals with Down syndrome to be in a moderate heart rate zone due to poorer functioning cardiovascular system (20).

### Post-resistance Training Intervention Test Visits

Participants completed 2 post resistance training intervention test visits 4–7 days after resistance training intervention (post-testing 1a and 1b). Post-testing visit 1a (cognitive/mood) was identical to the baseline visits 1 and 2a. Post-testing visit 1b (physical/motor) was identical to baseline 2b.

### Diet, Nutrition, and Exercise

With the aid of their parental/guardian/carer, participants recorded both drinks and food consumed 2 days prior to baseline visit 1a. To reduce external confounding factors, the participant replicated that diet 2 days prior to the second baseline visits (2a and 2b) and the post resistance exercise training test visits (1a and 1b). Additionally, participants were asked to maintain their regular diet and physical activity routine throughout the resistance training intervention, except for the individual resistance training sessions for the study.

### Motor Function Testing

The TGMD-2 was used to assess gross motor functioning, which assesses locomotor and object control motor skills such as a short sprint, gallop, shuffle, stationary basketball dribble, catch a baseball, etc. (18). Each task was individually explained and shown to the participant before s/he attempted, giving guidance cues as necessary.

### Cognitive Battery

Participants performed the validated computer-based battery, Arizona Cognitive Test Battery for Down syndrome (21). This testing battery consisted of several domains of CANTAB computerized testing, tracks assessment, and index finger tapping. The subjects were familiarized with this cognitive battery during familiarization visit 1 (visit 1). The battery shows sensitivity to within-sample differences and has specific correlates with brain function, along with being applicable to a wide range of severity of Down syndrome. Again as noted previously, Baseline testing showed stability with high test retest reliability of all measures analyzed for inclusion in this investigation.

### Survey Questionnaires

The same parental guardian participated in the questionnaires for all three cognitive testing visits assessing their child's perception of their child's intellectual ability during the previous 2 weeks (i.e., baseline 1a, 2a, and post-testing 1a). The cognitive-based questionnaires were the Cognitive Scale for Down Syndrome and the modified Behavioral Rating Inventory of Executive Function – Preschool Version questionnaire; both used to assess several domains of cognitive function (22). Additionally, the carer/parental guardian of the participant filled out a questionnaire relating to the recent mood status of the participant (Mood and Feelings Questionnaire: Long Version) to assess the variations of the mood of the participant.

The NiSonger Child Behavior Rating Form and the Scales of Independent Behavior-Revised (SIBR) Questionnaire were used to assess positive and negative behavior patterns subjectively. The Child Eating Behavior Questionnaire (CEBQ) (modified) was used to subjectively assess the participant's eating habits by assessing types of food/drink typically eaten, speed of eating, habits once full, etc. (23). The Social Communication Questionnaire (SCQ) assessed social awareness, language expression, interpretation of body language, etc. (24). Lastly, participants and parents/guardians answered an open-ended questionnaire assessing different areas and opinions related to resistance training at the full completion of the study (post-testing 1b).

### Flexibility, Physical Strength, DXA Scan

Participants completed a comprehensive physical fitness assessment, including muscular strength, muscular endurance, flexibility, and body composition. Following a brisk 5-min warm-up walk, flexibility was assessed via the sit-and-reach test (Pro Healthcare Products Park City, UT, USA). Muscular strength was assessed using 6-repetition maximum testing for the leg press (Plyo Press; Athletic Republic, Park City, UT) and bench press (Elite FTS, London, OH), as well as the 30-s chair sit-to-stand test (25). Muscular endurance was assessed using 30-s of push-ups (i.e., men full regulation toe push-ups; women modified) and 30-s of sit-ups (13). A dual-energy x-ray absorptiometry (DXA) whole-body scan was used to assess the body composition of each participant (GE Lunar IDxa; Encore Software). A pregnancy test was done via urine analysis before the DXA scans only for females to confirm that the woman was not pregnant. This scan was performed at baseline 2b and post-testing visit 1b.

### Statistical Analyses

Means and standard error of the means were calculated for each variable, and differences were assessed with parametric statistics. Data were analyzed using SPSS version 25 (IBM, Armonk, NY). Paired samples *t*-tests were used to assess differences between pre-to post-training intervention with Bonferroni corrections for alpha inflation. Baseline stability showed that all measures used in this study had ICCRs of *P* ≥ 0.75. Using the nQuery Advisor software (Statistical Solutions, Saugus, MA), it was determined that statistical power to defend the 0.05 alpha level of significance with a Cohen probability level of at least 0.75 was observed for each dependent variable. The significance in this study was set at *p* ≤ 0.05. All statistical assumptions for the statistical techniques were met. Additionally, effect sizes (ES) were calculated to assess the magnitude of change (Cohen's d = [M1–M2] / SD pooled), with values of 0.2, 0.5, and 0.8 considered small, medium, and large, respectively (26).

## Results

The primary findings of this study showed that a 10-week progressive resistance training program for adults with Down syndrome resulted in improvements in motor skill, cognitive performance, flexibility, physical strength, lean mass, and mood.

### Cognitive Baseline Testing

Due to the large variability of genotype penetration to a particular phenotype, a double baseline testing scheme was utilized to ensure consistency of the cognitive measures. As noted before all variables showed solid ICCRs and baseline stability as needed for such cognitive measures analyzed in this study (21). Additionally, no significant differences between BL1a and BL2a for our cognitive variables were observed.

### Pre/Post Resistance Training Intervention

**Figure 2:**
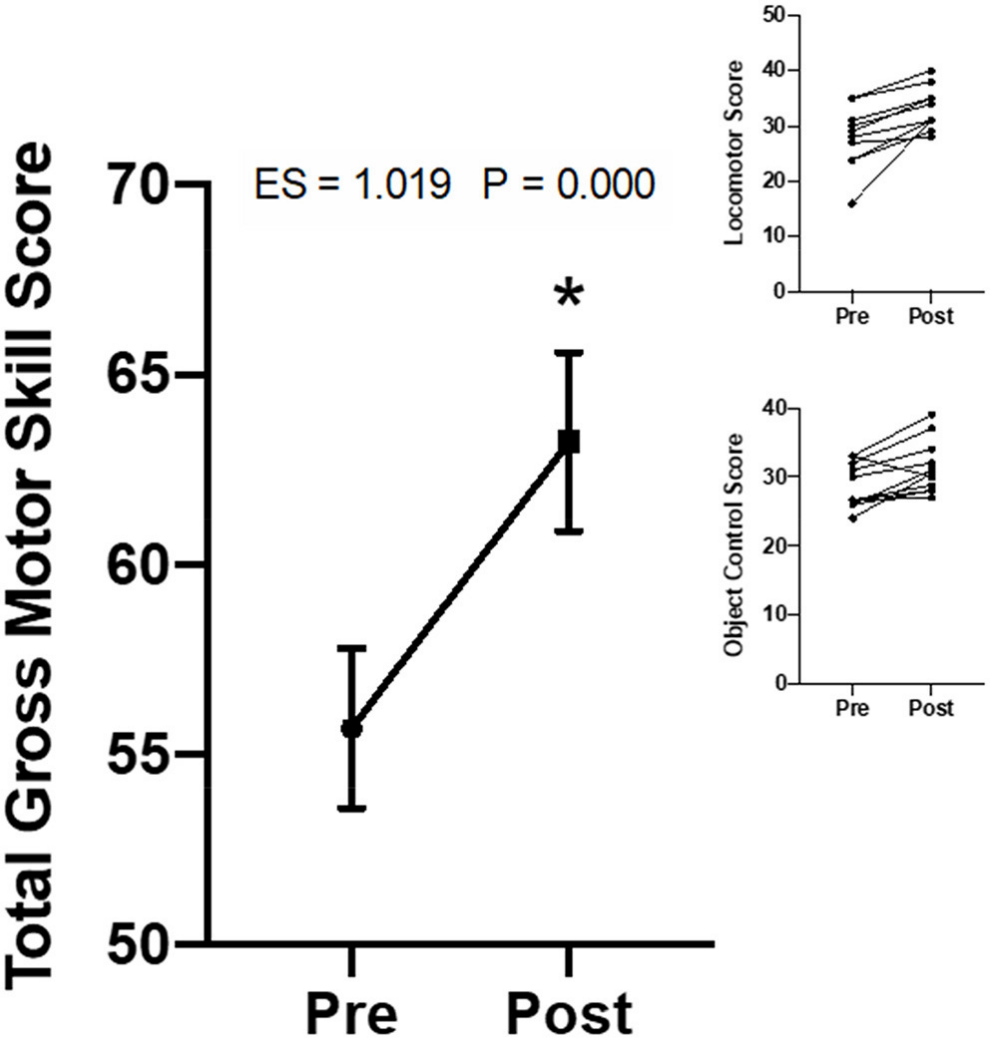
Change in the gross motor skill scores from the Total Gross Motor Development-version 2 (TGMD-2). Data are presented as means ± SEM. The figure inset shows the individual participant's mean scores for both locomotor skill and object control skill (*n* = 11). Every participant improved in all facets of motor skill (i.e., total, locomotor, object control) (*n* = 11). These data showed a 21.7% increase in overall motor skill (i.e., 29.3% increase in locomotor and 14.9% increase in object control). Individual data shown on the upper right inset panels. The “*” indicates a significant difference from pre-testing corresponding value.

Locomotor skill and object control skill are subsets of total gross motor skill (18). Total motor skill and subset metrics are shown in Figure 2. Participants significantly improved both locomotor skill (ES = 1.165, *p* = 0.001), object control skill (ES = 0.779, *p* = 0.008), and total gross motor function (ES = 1.019, *p* = 0.000) (Figure 2).

Cognitive performance metrics are presented in Table 2. The 10-week resistance training program significantly improved dominant hand 5 finger-tapping, forward total errors (i.e., examining visuospatial working memory), and shift behavior.

**Table 2:** Cognitive performance metrics (mean ± SD, *t*-test, *p*-value, and effect size).

	**Test assessment**	**Means** **+/- SD**		***p*-value**	**Effect size**
		**Pre**	**Post**		
KBITT-II	Non-verbal	19.0 ± 3.5	17.0 ± 4.6	0.116	0.189
	IQ composite	63.0 ± 9.4	58.1 ± 16.9	0.211	0.361
ACTB (NEPSY-II)	Tracks	6.7 ± 3.8	6.8 ± 4.3	0.878	0.116
	Dominant hand finger tapping	45.5 ± 30.9	28.7 ± 15.8	0.015^*^	0.681
**CANTAB testing**					
RTI	5-Choice Reaction Time	412.8 ± 115.2	389.4 ± 98.2	0.219	0.059
PAL	Total errors	36.3 ± 19.2	33.2 ± 16.1	0.503	0.176
SWM	Within errors	0.8 ± 0.9	1.5 ± 1.8	0.333	0.494
	Strategy	17.0 ± 1.5	17.5 ± 2.5	0.501	0.262
DMS	Percent correct	54.5 ± 13.8	57.0 ± 15.1	0.537	0.172
IED	Total adjusted errors	60.0 ± 17.2	77.5 ± 34.4	0.175	0.643
SSP	Forward total errors	13.5 ± 4.5	10.2 ± 3.3	0.019^*^	0.843
MTT	Incongruent cost	20.6 ± 51.4	41.5 ± 55.3	0.510	0.391
	Latency time	731.2 ± 206.3	769.4 ± 176.5	0.414	0.199
	Correct responses	100.5 ± 25.9	87.3 ± 25.3	0.250	0.516
	Incorrect responses	52.6 ± 25.9	66.1 ± 26.2	0.238	0.518

*One participant was excluded from the CANTAB portion of the analysis due being an extremely large outlier in every subset of the CANTAB tasks, largely skewing data (n = 10). This was the only set of testing that this participant was a large outlier on, therefore, was only excluded from these particular analyses*.

*MOT, Motor Screening Task; RTI, Reaction Time; PAL, Paired Associates Learning; SWM, Spatial Working Memory; DMS, Delayed Matching Sample; IED, Intra-Extra Dimensional Set Shift; SSP, Spatial Span; MTT, Multi-tasking Test; ES, Cohen's d effect size*.

* * = Significant difference from PRE (p **≤** 0.05)*.

**Figure 3:**
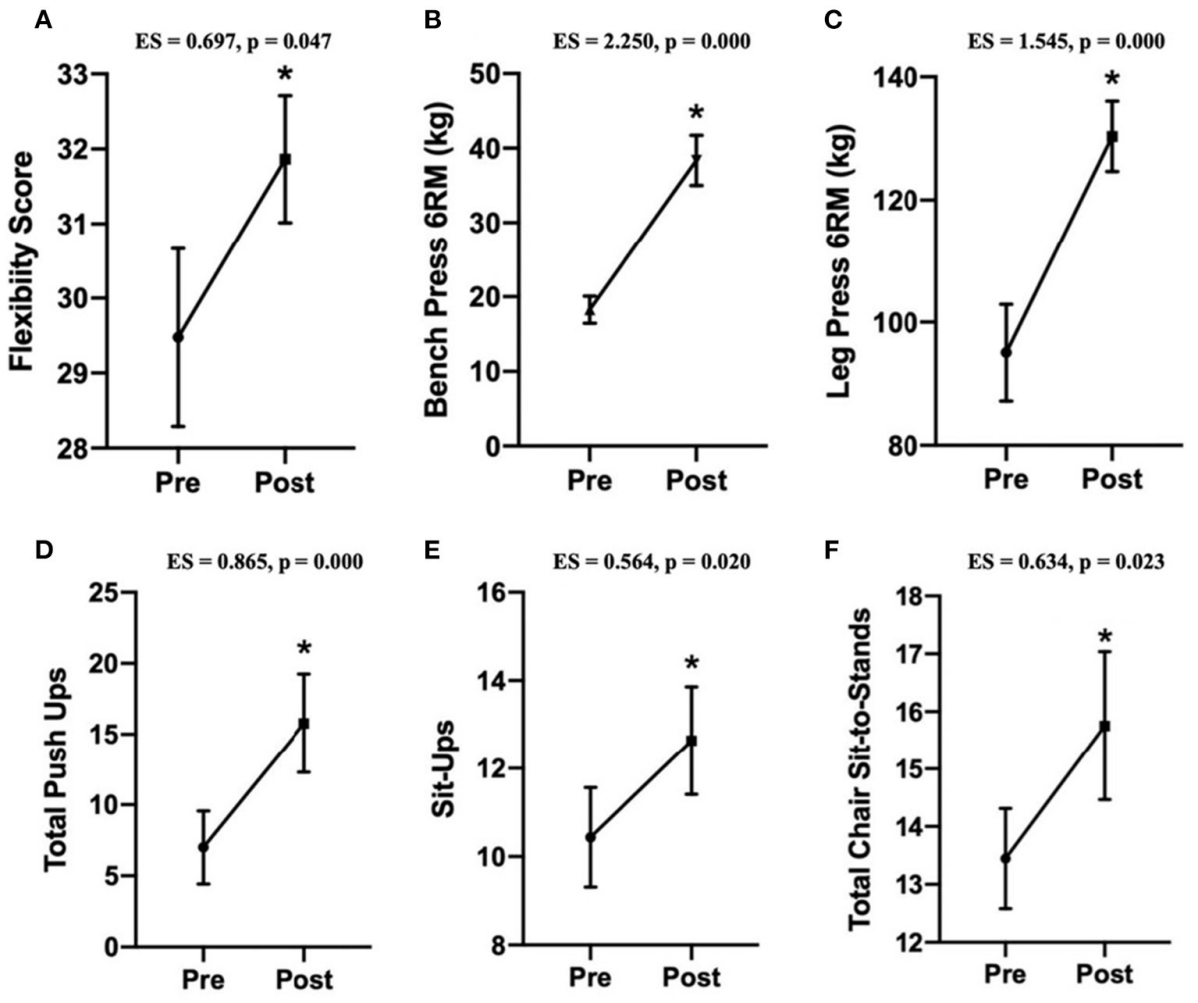
Data are presented as means ± SEM. The “*” indicates a significant difference from pre-intervention (*n* = 11). **(A)** Change in the flexibility score from the sit-and-reach flexibility test. **(B)** Change in the bench press 6 RM test. **(C)** Change in the lying leg press 6 RM test. **(D)** Change in the modified 30-s push-up test. **(E)** Change in the lying 30-s full sit-up test. **(F)** Change in the 30-s chair sit-to-stand test.

Physical fitness metrics are presented in Figure 3. This intervention significantly improved sit-and-reach flexibility, 6 RM barbell bench press, 6 RM supine leg press, 30-s push-ups, 30-s sit-ups, and 30-s chair sit-to-stand metrics.

With the 10 wks of resistance training there were no significant changes in overall body mass (ES = 0.057, *p* > 0.05), body fat percentage (ES = 0.035, *p* > 0.05), fat mass (ES = 0.007, *p* > 0.05), visceral fat mass (ES = 0.001, *p* > 0.05), or bone density (ES = 0.225, *p* > 0.05). However, there was a significant increase in lean body mass post-resistance training intervention (ES = 0.122, *p* < 0.05).

**Figure 4:**
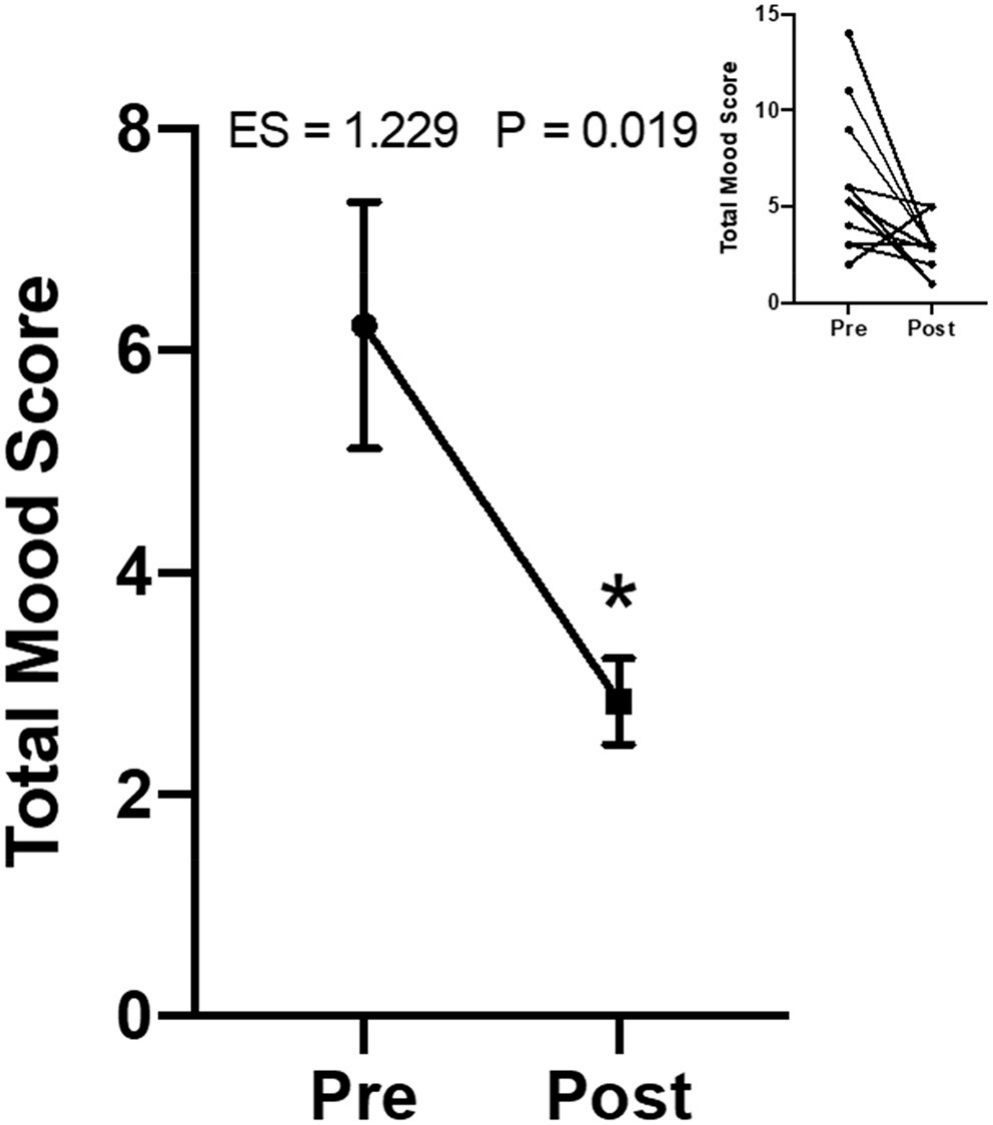
Change in the mood disturbance score from the Mood and Feelings Questionnaire parental survey (*n* = 11). Data are presented as means ± SEM. Individual data on upper right panel inset. The “*” indicates a significant difference from pre-intervention.

There was also a significant overall decrease in mood disturbances/unhappiness post-resistance training intervention (ES = 1.229, *p* < 0.05). Trait mood was improved largely after this program, showing decreased mood disturbances by 48.8% (Figure 4).

Parental questionnaire metrics regarding behavior, diet and social variables are shown in Table 3.

**Table 3:** Parental questionnaires metrics (mean ± SD, *t*-test, *p*-value, and effect size).

	**Test assessment**	**Means** **+/- SD**		***p*-value**	**Effect size**
		**Pre**	**Post**		
CS-DS	Total cognitive function	83.8 ± 12.6	82.7 ± 12.8	0.698	0.085
	Executive function	45.3 ± 6.2	45.8 ± 6.8	0.538	0.079
	Memory	26.1 ± 3.0	25.0 ± 3.4	0.094	0.335
	Language	11.7 ± 2.1	12.2 ± 2.4	0.366	0.194
NiSonger child behavior rating form	Social interactions	19.5 ± 4.9	20.2 ± 4.9	0.532	0.141
SIB-R	Shift Behavior	17.3 ± 2.8	15.9 ±2.8	0.016^*^	0.505
	Adaptive & Maladaptive Behavior	88.5 ± 12.9	93.3 ± 6.6	0.122	0.472
SCQ	Social communication	8.4 ± 1.6	7.7 ± 2.9	0.381	0.302
CEBQ	Emotional overeating	7.1 ± 1.4	7.3 ± 2.0	0.813	0.094
	Enjoyment of food	16.5 ± 2.0	16.3 ± 2.6	0.729	0.102
	Desire to drink	7.9 ± 1.3	7.3 ± 1.2	0.108	0.495
	Satiety responsiveness	12.3 ± 1.4	12.7 ± 1.4	0.321	0.284
	Slowness in eating	12.0 ± 1.7	11.5 ± 1.7	0.060	0.303
	Emotional under-eating	7.0 ± 1.7	7.7 ± 1.1	0.082	0.502
	Food fussiness	15.7 ± 3.1	16.0 ± 3.9	0.729	0.082
	Food responsiveness	14.2 ± 2.4	10.3 ± 3.5	0.002^*^	1.282

*SIB-R, Scales of Independent Behavior-Revised; CS-DS, Cognitive Scale for Down Syndrome; BRIEF-P, Behavioral Rating Inventory of Executive Function – Preschool; SCQ, Social Communication Questionnaire; CEBQ, Child Eating Behavior Questionnaire (CEBQ) (modified)*.

* *= Significant difference from PRE (p ≤ 0.05)*.

Lastly, when the participants and parents were asked about the overall resistance training program, overarching themes were commonly used throughout the questionnaire. The main themes include increased self-confidence, increased ability to complete activities of daily living, and increased interest in exercise, physical activity, nutrition, and overall health.

## Discussion

The primary findings of this 10-week resistance training program for young adults with Down syndrome were positive effects observed on motor performance, cognitive function, physical performance, and structural changes in the body (i.e., lower body flexibility, physical strength, physical endurance, lean body mass). The most compelling findings were improvements in motor proficiency and aspects of cognitive function. These results indicate a positive impact of individualized progressive heavy resistance training for young adults with Down syndrome.

This resistance training program significantly improved overall gross motor skills, which consists of specific locomotor (i.e., actions propelling an individual from one place to another) and object control motor skills (i.e., abilities to move objects in an accurate and controlled way) subsets (18). Secondly, there was an increase in dominant hand sensorimotor skills. General resistance training involves many gross motor skill learning elements such as balance, coordination, and power. Initial strength gains following initiation of a resistance training program are commonly attributed to neural adaptations, which may be responsible for the improvements seen in motor proficiency. Improvements in motor function would significantly impact an individual's quality of life. Structural differences (i.e., short neck, small hands and feet, shortened height) for individuals with Down syndrome often leads to impaired motor learning at a young age that negatively impact motor functioning and lead to decreased independence into adulthood (11, 27, 28). Motor skill and coordination are critical factors for activities of daily living (i.e., making dinner, walking up and down steps, folding laundry, showering, using the restroom, etc.), confirming the significance of such improvements found with resistance training in this study.

This study's resistance training intervention significantly improved motor skills which implies favorable cognitive change due to the strong, established link between cognitive function and motor proficiency (27, 28). The frontal lobe of the brain of individuals with Down syndrome tends to have the longest development period, which typically increases the magnitude for change (29), potentially causing a more significant positive impact through exercise (i.e., prefrontal cortex M1 region of the frontal lobe area of the brain). Although there is little literature looking specifically into adults with Down syndrome and specific improvements in motor skill, the literature has already shown the well-established role of the frontal lobe primary motor cortex (M1) within motor skill adaptations. This has been shown to explicitly play a critical role in the retention of motor learning which is typically a consequence of repetitive performance of the same movement pattern relying on synaptic efficacy, potentially resulting in a change in corticospinal and dendritic spinal excitability in the typically developed population (30–32).

The initial motor skill improvements demonstrated in this study could potentially be mediated by the M1 frontal lobe region of the brain has made adjustments based on errors made throughout the motor learning process within the repetitive task stimulus. This indicates that in individuals with Down syndrome, a long-term potentiation-like process in the M1 mediates the initial learning of simple motor skills (28, 30). This confirms the critical role that the M1 region of the brain has in forming the initial model for motor skills, more importantly, that the M1 region solidifies the maintenance of that learned skill and stores the sequencing of the simple motor learning tasks to contribute highly to motor sequence learning, for complex motor tasks (28, 30), especially as observed within our study population. During this intervention, repeated strength training movements, from simple movements requiring neuromuscular activation to complex movements requiring integrated task-specific timing and coordination, the exercise program potentially increased synaptic efficacy to increase motor retention, improving overall motor skill functions over time. The early motor skill adaptations could also be due to plastic changes and cerebellar excitability throughout the cerebellum (17, 30, 33, 34). An internal sensorimotor map for each skill is developed when motor learning that has been developed is engaged. However, the error-based mechanisms, shown through dynamic plastic changes in the cerebellum, appear to have less of a role later as a task training progresses both behaviorally and physiologically (17, 30, 35). This pathway between the cerebellum and M1 is critical for motor learning during movement preparation and movement to form the motor skills (17). With our resistance training program these neural connections between the cerebellum and M1 area of the brain appear to have been strengthened in order to allow for learning of various new exercise movements. Thus for our adults with Down syndrome, more complex motor skills were be learned over time through enhanced M1 neural interconnections.

Our results showed an improvement in areas of executive functioning, i.e., visuospatial working memory, such that subjects had a decrease in total forward errors assessed using the Spatial Span (SSP) test. The frontal lobe (i.e., location of M1) and visuospatial memory also have a strong connection to each other in their function and role in the body (36) and tend to be developed later and operate at a lower level for adults with Down syndrome than observed in the general adult population (7, 29). This study population lies in the mild to moderate intellectual level in individuals with Down syndrome (37). The improvements observed from this test indicate resistance training may positively impact cognitive performance within the mild to moderate severity of Down syndrome.

The significant improvements in visuospatial working memory found in this study align with current literature assessing resistance training interventions in typically cognitively developed adults (38, 39) and individuals who are intellectually handicapped (6, 39). Additionally, the decrease in time for the dominant hand 5 finger-tapping shows potential in the role of resistance exercise on the dominant side neurological function. Although these are different subsets of the population, many of the same mechanisms can apply to individuals with Down syndrome, specifically that resistance training positively impacts the frontal region of the brain, as supported by the favorable improvement on both motor skill and working memory; both primarily housed in the frontal lobe of the brain (40). Individuals with Down syndrome may not be functioning optimally with functional connectivity regarding communication between several brain regions (29, 36), which supports the importance of the two most likely explanations being long-range connectivity and top-down signaling functionalities. There is strong functional connectivity between the fusiform face area and the prefrontal cortex, which supports higher-order functioning and maintenance of sensory perception (36), along with evidence implying that top-down signaling through synaptic aftereffects in recurrent circuits/synchronous oscillations between neuronal cells can bias the likelihood of relevant task-relevant information in a competitive system (36). As mentioned above, the long-range connectivity between the cerebellum, located just near the fusiform face area, and M1 is critical with motor skill processing and retention (17) within the Down syndrome population. As seen in this study, if resistance training impacts both of these brain regions, improvements in working memory may become more operational.

We found significant improvement in shift behavior after the intervention. Previously, literature has shown that resistance and aerobic exercise improve shift behavior chronically, as measured in generally healthy individuals (41). The positive change in shift/problem-solving behavior could be due to increased attentional allocation, associating this change in attention to later stages of mental processing (41), specifically storing and retrieving processing. This intervention may have aided everyday life changes and problems solving that arise from this favorable shift behavior change, positively impacting daily life.

The drastic significant changes in gross and fine motor skills and change in shift behavior imply some initial changes in cognitive performance resulting from resistance training. There are well-established lines of connection between motor skill, cognition, and brain development through prior research studies (17, 30–35). Due to the extreme change in gross motor skill, adaptive neural tissue and interneuron connections appear plausible, showing the positive impact of resistance training in our study population.

Adults with Down syndrome have above healthy average values of flexibility than a generally healthy adult due to differences in connective tissue, creating greater hypermobile joints (42). Therefore, small increases in flexibility for this population of adults, as shown in this study, was an impressive finding. This increase in flexibility is still beneficial. It can help alleviate and/or avoid lower back pain that can come with a lack of flexibility in these areas throughout aging, without any additional injury risk (43).

We anticipated that with periodized progressive heavy resistance training, there would be significant increases in full-body strength (i.e., upper body, lower body) and local muscular endurance (i.e., abdominal and push-ups). The strength improvements in the upper and lower body observed from this resistance training program are likely both neural (i.e., intramuscular and intermuscular coordination) and muscular (i.e., hypertrophy due to significant increases in lean body mass) adaptive components. Neural factors tend to contribute most to muscular adaptations in the beginning stages of a strength training program due to an increase in motor unit firing rate and/or change in the pattern of motor unit activation (44), with gradual increases in the hypertrophic factors as the training continues (45). This causes a more efficient motor unit activation, leading to increased strength early on, as witnessed in this study. Individuals with Down syndrome have a high prevalence of hypotonia (i.e., a global descriptive qualitative view of muscles laxity and movement) and variations in connective tissue that lead to issues with muscle weakness, joint instability (46), and increased risk of injury (47). This population is also at a higher risk for muscle weakness in general (10, 46), along with most job prospects being labor-intensive (48). Increasing muscular strength, thereby increasing the strength around joints, creates better joint stability, helps one perform more labor-intensive jobs, and minimizes the risk of injury.

The significant increase in lean body mass was likely caused by the muscle contractile units and process of protein synthesis, i.e., likely hypertrophy, especially with the inclusion of heavier loading periodized into the training program. Evidence shows the benefits of resistance training in the general adult population (i.e., increased muscular strength, hypertrophy, and lean muscle mass) (46). Based on our findings, many of those same benefits are translatable to adults with Down syndrome. We postulate that the larger lean tissue mass increase for some participants than others was likely due to differences in muscles' use during everyday activities. Heavier loading also allows hypertrophic factors to play a larger role in strength improvements, increasing cross-sectional muscle fiber sizes. Although still within a healthy limit, adults with Down syndrome tend to have higher creatine values than a typically developed individual (49), potentially increasing the magnitude of change for lean body mass by tolerating higher volumes and loads.

An important and often forgotten element in many studies is the intervention's impact on the parents/guardians. This group reported fewer mood disturbances following this resistance training program, reflecting an overall improvement in trait mood. Mikkelsen et al. (50) found overall general mental health benefits due to exercise for generally healthy adults, i.e., resistance training, which translates to adults with Down syndrome based on our current findings. Prior research has implicated possible mechanisms for these changes as resistance training increases circulating plasma endorphin and hormone values (50), increases thermogenic state (i.e., brain regions) to induce relaxation and decrease anxiety (50), increases mitochondrial function, directly impacting neurogenesis and neuroplasticity (50), and attenuates the hypothalamic-pituitary-adrenal axis such to counteract the high values of cortisol and corticotrophin-releasing hormone and compromised function of glucocorticoids (50). These physiological mechanisms then may mediate the decrease in overall mood disturbances, improving overall mood.

Additionally, there was a decrease in negative food responsiveness, meaning participants tended to have a healthier relationship with food post-resistance training program while being more intentional with the quantity and quality of food eaten. Social support is a significant indicator of positive health behavior outcomes, especially with individuals with Down syndrome (51). Participants were in an encouraging environment, essentially providing social support throughout the study and becoming aware of the quality and quantity of food they were ingesting.

A hundred percent of subjects mentioned increased self-confidence after the intervention was completed, along with a peaked interest in overall health (i.e., exercise and diet) during the post-intervention open-ended survey. The consistent increase in self-confidence is consistent with one of Bandura's Social-Cognitive Theory (SCT) constructs that rely heavily on self-efficacy and outcome expectations (52). It is highly possible that participants in this study felt more aware of their choices and their ability to control their health and recognize the consequences of their choices, which aligns with the SCT (52) for individuals with Down syndrome. Participants in this resistance training intervention were able to develop a strong social connection with the personal trainers while developing a positive relationship with resistance training due to the individualization of the program. This social connection between the trainer(s) and participant likely spurred the encouragement needed to be more aware of healthy choices and enjoy the exercise program. This social aspect also helped alleviate common barriers for this population.

In summary, the findings of this investigation reveal the positive benefits of resistance training for adults with Down syndrome. The salient results from this study were that beyond the expected gains in strength and endurance with resistance training were the improvements in gross motor skill functioning and mood improvements. The cognitive performance also showed beneficial change, specifically regarding executive function and frontal lobe activity. This is the first study to examine and show positive cognitive and overall motor skill performance changes in response to a periodized progressive heavy resistance training program in young adults with Down syndrome. This connection should be further investigated since motor skill functioning has a cognitive component. These novel findings also suggest future research to examine further the relationship between executive function/frontal lobe activity and resistance training and how this may impact mood and everyday quality of life. Secondly, this study confirmed the strength and lean body mass benefits of resistance training for young adults with Down syndrome. This study is one of the first of its kind and contributes important new findings to the current literature base and brings to the forefront in exercise science the importance of such research to be funded and published to support evidence-based data for the use of this modality. Thus, we recommend that adults with Down syndrome consistently participate in resistance training programs. We realize our limitations but hope this study stimulates future studies that have large n sizes, age groups and separate experimental groups of men and women. Additionally longer resistance training programs also need to be studied as to the improvements observed in the current investigation to determine various timelines of change for individual participants.

## Data Availability Statement

The raw data supporting the conclusions of this article will be made available by the authors, without undue reservation.

## Ethics Statement

The studies involving human participants were reviewed and approved by the Ohio State University's Institutional Review Board for the use of human subjects in research. The patients/participants provided their written informed consent to participate in this study.

## Author Contributions

Each author was involved with the important parts of the experiment including conception of design of the research. EP, WK, JV, BF, and CM: data collection. EP, MK, LC, and BS: data analysis: EP, WK, BF, JV, and CM: interpretation of the data. All authors: drafted the paper. EP, WK, JV, CM, MK, LC: critical revision of draft. WK, JV, CM, RN, and KH: final approval. All authors contributed to the article and approved the submitted version.

## Funding

This study was supported in part by a doctoral student grant from the National Strength and Conditioning Association and the Ohio State University investigator and laboratory funds.

## Conflict of Interest

The authors declare that the research was conducted in the absence of any commercial or financial relationships that could be construed as a potential conflict ofinterest.

## Publisher's Note

All claims expressed in this article are solely those of the authors and do not necessarily represent those of their affiliated organizations, or those of the publisher, the editors and the reviewers. Any product that may be evaluated in this article, or claim that may be made by its manufacturer, is not guaranteed or endorsed by the publisher.

## References

[1] Prevention CfDCa. Occurrence of Down syndrome in the United States. 2017.

[2] Shields N Dodd K. A systematic review on the effects of exercise programmes designed to improve strength for people with down syndrome. Physical Therapy Reviews. (2013) 9:109–15. 10.1179/108331904225005043

[3] Fortea J Zaman SH Hartley S Rafii MS Head E Carmona-Iragui M. Alzheimer's disease associated with Down syndrome: a genetic form of dementia. Lancet Neurol. (2021) 20:930–42. 10.1016/S1474-4422(21)00245-3 34687637 PMC9387748

[4] Hartley D Blumenthal T Carrillo M DiPaolo G Esralew L Gardiner K. Down syndrome and Alzheimer's disease: common pathways, common goals. Alzheimers Dement. (2015) 11:700–9. 10.1016/j.jalz.2014.10.007 25510383 PMC4817997

[5] Hithersay R Hamburg S Knight B Strydom A. Cognitive decline and dementia in down syndrome. Curr Opin Psychiatry. (2017) 30:102–7. 10.1097/YCO.0000000000000307 28009725

[6] Chupel MU Direito F Furtado GE Minuzzi LG Pedrosa FM Colado JC. Strength training decreases inflammation and increases cognition and physical fitness in older women with cognitive impairment. Front Physiol. (2017) 8:377. 10.3389/fphys.2017.00377 28659812 PMC5467003

[7] Ptomey LT Szabo AN Willis EA Gorczyca AM Greene JL Danon JC. Changes in cognitive function after a 12-week exercise intervention in adults with Down syndrome. Disabil Health J. (2018) 11:486–90. 10.1016/j.dhjo.2018.02.003 29501470 PMC6005720

[8] Nordgren B Bäckström L. Correlations between muscular strength and industrial work performance in mentally retarded persons. Acta Paediat Scand Suppl. (1971) 217:5710. 10.1111/j.1651-2227.1971.tb05710.x 5289781

[9] Latella C Teo WP Harris D Major B VanderWesthuizen D Hendy AM. Effects of acute resistance training modality on corticospinal excitability, intra-cortical and neuromuscular responses. Eur J Appl Physiol. (2017) 117:2211–24. 10.1007/s00421-017-3709-7 28879576

[10] Agiovlasitis S Mendonca GV McCubbin JA Fernhall B. Prediction of energy expenditure during walking in adults with down syndrome. J Appl Res Intellect Disabil. (2018) 31(Suppl 1):151–6. 10.1111/jar.12392 28815878

[11] Rosety-Rodriguez M Bernardi M Elosegui S Rosety I Diaz AJ Rosety MA. A short-term resistance training circuit improved antioxidants in sedentary adults with down syndrome. Oxid Med Cell Longev. (2021) 2021:8811153. 10.1155/2021/8811153 33532037 PMC7840230

[12] Mendonca GV Pereira FD Fernhall B. Effects of combined aerobic and resistance exercise training in adults with and without down syndrome. Arch Phys Med Rehabil. (2011) 92:37–45. 10.1016/j.apmr.2010.09.015 21187203

[13] Lewis CL Fragala-Pinkham MA. Effects of aerobic conditioning and strength training on a child with down syndrome: a case study. Pediatric Physical Therapy. (2005) 17:30–6. 10.1097/01.PEP.0000154185.55735.A0 16357655

[14] Fernhall B. Physical fitness and exercise training in individuals with MR. Med Sci Sports Exe. 1993:442–50. 10.1249/00005768-199304000-00006 8479298

[15] Suomi R. Self-directed strength training- Its effect on leg strength in men with mental retardation. Arch Phys Med Rehabil. (1998) 79:14. 10.1016/S0003-9993(98)90014-4 9523786

[16] Center UDAsD. UC Davis Protocol Deciding Assent Versus Consent. Oakland, CA: The Regents of the University of California (2002).

[17] Spampinato DA Block HJ Celnik PA. Cerebellar-M1 connectivity changes associated with motor learning are somatotopic specific. J Neurosci. (2017) 37:2377–86. 10.1523/JNEUROSCI.2511-16.2017 28137969 PMC5354349

[18] Ulrich D. Test of Gross Motor Development. 2nd ed. Austin, TX: Proed (2000).

[19] Eberhard Y Eterrodossi J Debû B. Biological changes induced by physical activity in individuals with Down's syndrome. Adapted Physical Activity Quarterly. (1997) 14:166–75. 10.1123/apaq.14.2.166

[20] Vis JC De Bruin-Bon HA Bouma BJ Huisman SA Imschoot L van den Brink K. Adults with Down syndrome have reduced cardiac response after light exercise testing. Neth Heart J. (2012) 20:264–9. 10.1007/s12471-012-0254-1 22331518 PMC3370088

[21] Edgin JO Anand P Rosser T Pierpont EI Figueroa C Hamilton D. The Arizona cognitive test battery for down syndrome: test-retest reliability and practice effects. Am J Intellect Dev Disabil. (2017) 122:215–34. 10.1352/1944-7558-122.3.215 28452581 PMC6215707

[22] Startin CM Rodger E Fodor-Wynne L Hamburg S Strydom A. Developing an informant questionnaire for cognitive abilities in down syndrome: the cognitive scale for Down syndrome (CS-DS). PLoS ONE. (2016) 11:e0154596. 10.1371/journal.pone.0154596 27153191 PMC4859552

[23] Wardle J Guthrie C Sanderson S Rapoport L. Development of the children's eating behaviour questionnaire. J Child Psychol Psych. (2001) 42:963–70. 10.1111/1469-7610.00792 11693591

[24] Chesnut SR Wei T Barnard-Brak L Richman DM. A meta-analysis of the social communication questionnaire: screening for autism spectrum disorder. Autism. (2017) 21:920–8. 10.1177/1362361316660065 27503464

[25] Shields N Taylor N Fernhall B. A study protocol of a randomised controlled trial to investigate if a community based strength training programme improves work task performance in young adults with Down syndrome. BMC Pediatrics. (2010) 10:17. 10.1186/1471-2431-10-17 20334692 PMC2858133

[26] Cohen J. Statistical Power Analysis for the Behavioral Sciences. Hillsdale, NJ: L Erlbaum Associates (1988).

[27] Alesi M Battaglia G Pepi A Bianco A Palma A. Gross motor proficiency and intellectual functioning: a comparison among children with Down syndrome, children with borderline intellectual functioning, and typically developing children. Medicine. (2018) 97:e12737. 10.1097/MD.0000000000012737 30313077 PMC6203563

[28] Hartman E Houwen S Scherder E Visscher C. On the relationship between motor performance and executive functioning in children with intellectual disabilities. J Intellect Disabil Res. (2010) 54:468–77. 10.1111/j.1365-2788.2010.01284.x 20537052

[29] Edgin JO. Cognition in Down syndrome: a developmental cognitive neuroscience perspective. Wiley Interdiscip Rev Cogn Sci. (2013) 4:307–17. 10.1002/wcs.1221 26304208

[30] Spampinato D Celnik P. Deconstructing skill learning and its physiological mechanisms. Cortex. (2018) 104:90–102. 10.1016/j.cortex.2018.03.017 29775838

[31] Galea JM Vazquez A Pasricha N Orban de. Xivry JJ, Celnik P. Dissociating the roles of the cerebellum and motor cortex during adaptive learning: the motor cortex retains what the cerebellum learns. Cerebral Cortex. (2011) 21:1761–70. 10.1093/cercor/bhq246 21139077 PMC3138512

[32] Harms KJ Rioult-Pedotti MS Carter DR Dunaevsky A. Transient spine expansion and learning-induced plasticity in layer 1 primary motor cortex. J Neurosci. (2008) 28:5686–90. 10.1523/JNEUROSCI.0584-08.2008 18509029 PMC2793590

[33] Kleim J Pipitone M Czerlanis C Greenough W. Structural stability within the lateral cerebellar nucleus of the rat following complex motor learning. Neurobiol Learn Mem. (1998) 69:290–306. 10.1006/nlme.1998.3828 9707491

[34] Yang Y Lisberger SG. Role of plasticity at different sites across the time course of cerebellar motor learning. J Neurosci. (2014) 34:7077–90. 10.1523/JNEUROSCI.0017-14.2014 24849344 PMC4028490

[35] Schlerf JE Galea JM Bastian AJ Celnik PA. Dynamic modulation of cerebellar excitability for abrupt, but not gradual, visuomotor adaptation. J Neurosci. (2012) 32:11610–7. 10.1523/JNEUROSCI.1609-12.2012 22915105 PMC3435878

[36] D'Esposito M Postle BR. The cognitive neuroscience of working memory. Annu Rev Psychol. (2015) 66:115–42. 10.1146/annurev-psych-010814-015031 25251486 PMC4374359

[37] TestPrep-Online. KBIT Test Scores Explained. Tel-Aviv, IL: TestPrep-Online (2020).

[38] Mavros Y Gates N Wilson GC Jain N Meiklejohn J Brodaty H. Mediation of cognitive function improvements by strength gains after resistance training in older adults with mild cognitive impairment: outcomes of the study of mental and resistance training. J Am Geriatr Soc. (2017) 65:550–9. 10.1111/jgs.14542 28304092

[39] Chang YT. Physical activity and cognitive function in mild cognitive impairment. ASN Neuro. (2020) 12:1182. 10.1177/1759091419901182 31948261 PMC6970452

[40] Penhune VB Steele CJ. Parallel contributions of cerebellar, striatal and M1 mechanisms to motor sequence learning. Behav Brain Res. (2012) 226:579–91. 10.1016/j.bbr.2011.09.044 22004979

[41] Wu CH Karageorghis CI Wang CC Chu CH Kao SC Hung TM. Effects of acute aerobic and resistance exercise on executive function: An ERP study. J Sci Med Sport. (2019) 22:1367–72. 10.1016/j.jsams.2019.07.009 31445953

[42] Parker A James B. Age changes in the flexibility of Down's syndrome children. J Ment Defic Res. (1985) 29:207–18. 10.1111/j.1365-2788.1985.tb00330.x 2933522

[43] Sadler SG Spink MJ Ho A De Jonge XJ Chuter VH. Restriction in lateral bending range of motion, lumbar lordosis, and hamstring flexibility predicts the development of low back pain: a systematic review of prospective cohort studies. BMC Musculoskelet Disord. (2017) 18:179. 10.1186/s12891-017-1534-0 28476110 PMC5418732

[44] Gabriel D Kamen G Frost G. Neural adaptations to resistive exercise mechanisms and recommendations for training practices. Sports Med. (2006) 36:133–49. 10.2165/00007256-200636020-00004 16464122

[45] Moritani T DeVries H. Neural factors versus hypertrophy in the time course of muscle strength gain. Am J Phys Med. (1970) 38:115–30. 453338

[46] Diaz AJ Rosety I Ordonez FJ Brenes F Garcia-Gomez N Castejon-Riber C. Effects of resistance training in muscle mass and markers of muscle damage in adults with down syndrome. Int J Environ Res Public Health. (2021) 18:8996. 10.3390/ijerph18178996 34501582 PMC8431092

[47] Morris A Vaughan S Vaccaro P. Measurements of neuromuscular tone and strength in down's syndrome children. J Ment Defic Res. (1982) 26:41–6. 10.1111/j.1365-2788.1982.tb00127.x 6210779

[48] Cowley PM Ploutz-Snyder LL Baynard T Heffernan KS Jae SY Hsu S. The effect of progressive resistance training on leg strength, aerobic capacity and functional tasks of daily living in persons with Down syndrome. Disabil Rehabil. (2011) 33:2229–36. 10.3109/09638288.2011.563820 21446859

[49] Hestnes A Stovner L Husoy O Folling I Fougner K Staastad O. Hormonal and biochemical disturbances in Down's syndrome. J Ment Defic Res. (1991) 35:179–93. 10.1111/j.1365-2788.1991.tb01051.x 1833549

[50] Mikkelsen K Stojanovska L Polenakovic M Bosevski M Apostolopoulos V. Exercise and mental health. Maturitas. (2017) 106:48–56. 10.1016/j.maturitas.2017.09.003 29150166

[51] Mahy J Shields N Taylor NF Dodd KJ. Identifying facilitators and barriers to physical activity for adults with Down syndrome. J Intellect Disabil Res. (2010) 54:795–805. 10.1111/j.1365-2788.2010.01308.x 20712696

[52] Young MD Plotnikoff RC Collins CE Callister R Morgan PJ. Social cognitive theory and physical activity: a systematic review and meta-analysis. Obes Rev. (2014) 15:983–95. 10.1111/obr.12225 25428600

